# Circular RNA circ-NT5C2 acts as an oncogene in osteosarcoma proliferation and metastasis through targeting miR-448

**DOI:** 10.18632/oncotarget.22162

**Published:** 2017-10-30

**Authors:** Xunfa Liu, Yuanbo Zhong, Jifeng Li, Aijun Shan

**Affiliations:** ^1^ Department of Emergency, Second Clinical Medical College, Shenzhen People’s Hospital, Jinan University, Shenzhen, 518020, China; ^2^ College of Basic Medicine, Jinan University, Guangzhou, Guangdong, 510632, China

**Keywords:** osteosarcoma, circular RNA, circ-NT5C2, circRNA microarray, miR-448

## Abstract

Circular RNAs (circRNAs) are a type of endogenous noncoding RNA which have been verified to participate in numerous pathophysiological processes. However, the underlying role of circRNAs in osteosarcoma tissue is still unidentified. Our study aims to investigate the circRNA expression profiles in osteosarcoma tissue and investigate the physiological functions of circRNAs. Human circRNAs microarray analysis showed that 785 differently expressed circRNAs were distinguished in osteosarcoma tissue and adjacent non-tumor tissue with 2 fold change. Circ-NT5C2 was validated to be up-regulated expressed in 52 pairs of osteosarcoma tissue and cell lines. Furthermore, the enforced expression of circ-NT5C2 could act as a valuable diagnostic marker for osteosarcoma detection with AUC (area under the ROC curve) value of 0.753. Functional validation experiments verified that circ-NT5C2 silencing suppressed the proliferation and invasion, and promoted apoptosis of osteosarcoma cells *in vitro*. *In vivo*, circ-NT5C2 silencing inhibited the tumor growth. Bioinformatics analysis and rescue experiments indicated that circ-NT5C2 sponged miR-448, which was confirmed by luciferase reporter assay and RT-PCR assay. Overall, our study investigates the circRNAs expression profiles and determines the function of circ-NT5C2 in osteosarcoma tumorigenesis, which might serve as a novel therapeutic target of osteosarcoma patients.

## INTRODUCTION

Among the primary bone malignant tumors, osteosarcoma is one of the most common cancers in children and adolescents worldwide, acting as a leading cause of cancer mortality [1]. However, the overall survival rate of osteosarcoma patients remains pessimistic [2]. The metastases and relapse of osteosarcoma cells usually cause the poor prognosis and low 5-year survival rate [3]. Presently, the main method of diagnoses and therapies, tumor excision combined with adjuvant chemotherapy, could achieve limited effects [4]. Therefore, basis of in-depth understanding of molecular mechanisms involved in osteosarcoma tumorigenesis and progression, there is potential unidentified pathogenesis for the osteosarcoma carcinogenesis needing to discover, which might be the novel therapeutic targets for osteosarcoma [5].

Circular RNAs (circRNAs) are a class of noncoding RNA characterized by covalently closed circular loop with a lack of capability for protein coding, which play critical biological roles in carcinogenesis and development [6]. CircRNAs can be generated from exons or introns, forming exonic or intronic circRNAs [7]. Compared with traditional linear RNA, for example lncRNA, circRNAs are structured as a covalently closed loop without 5′ to 3′ polarity or polyadenylated tail [8]. Because circRNAs have the specific configuration, their physiological roles are increasing attaching attention.

Increasing evidences indicate that circRNAs play important roles in series of pathological and physiological process. For example, circular RNA circMTO1 suppresses HCC progression by acting as the sponge of oncogenic miR-9 to promote p21 expression [9]. In vascular endothelial cells, circRNA microarray analysis reveals that hsa_circ_0010729 regulates VECs proliferation and apoptosis via targeting miR-186/HIF-1α axis [10]. Besides, circRNAs might stably exist in tissue and peripheral blood, suggesting the potential role for biomarkers [11]. In lung adenocarcinoma, hsa_circ_0013958 is further confirmed to be up-regulated in the LAC tissues and act as potential novel biomarker [12].

In present study, we screen the circRNA expression profile in 4 pairs of osteosarcoma tissue samples and adjacent non-tumor tissue, and revealed that circ-NT5C2 was significantly up-regulated in osteosarcoma tissue. CircRNA circ-NT5C2 (hsa_circ_0092509, circBase, www.circbase.org/) located at chr10: 104850367-104850753 with 386 length and gene symbol NT5C2. The aim of this study is to characterize the circRNA expression profiles of osteosarcoma tissue and identify the physiological function of circ-NT5C2, thus providing new insight into the molecular signatures of osteosarcoma tumorigenesis.

## RESULTS

### Circular RNA microarray expression profiles of osteosarcoma tissue and adjacent normal tissue

**Figure 1:**
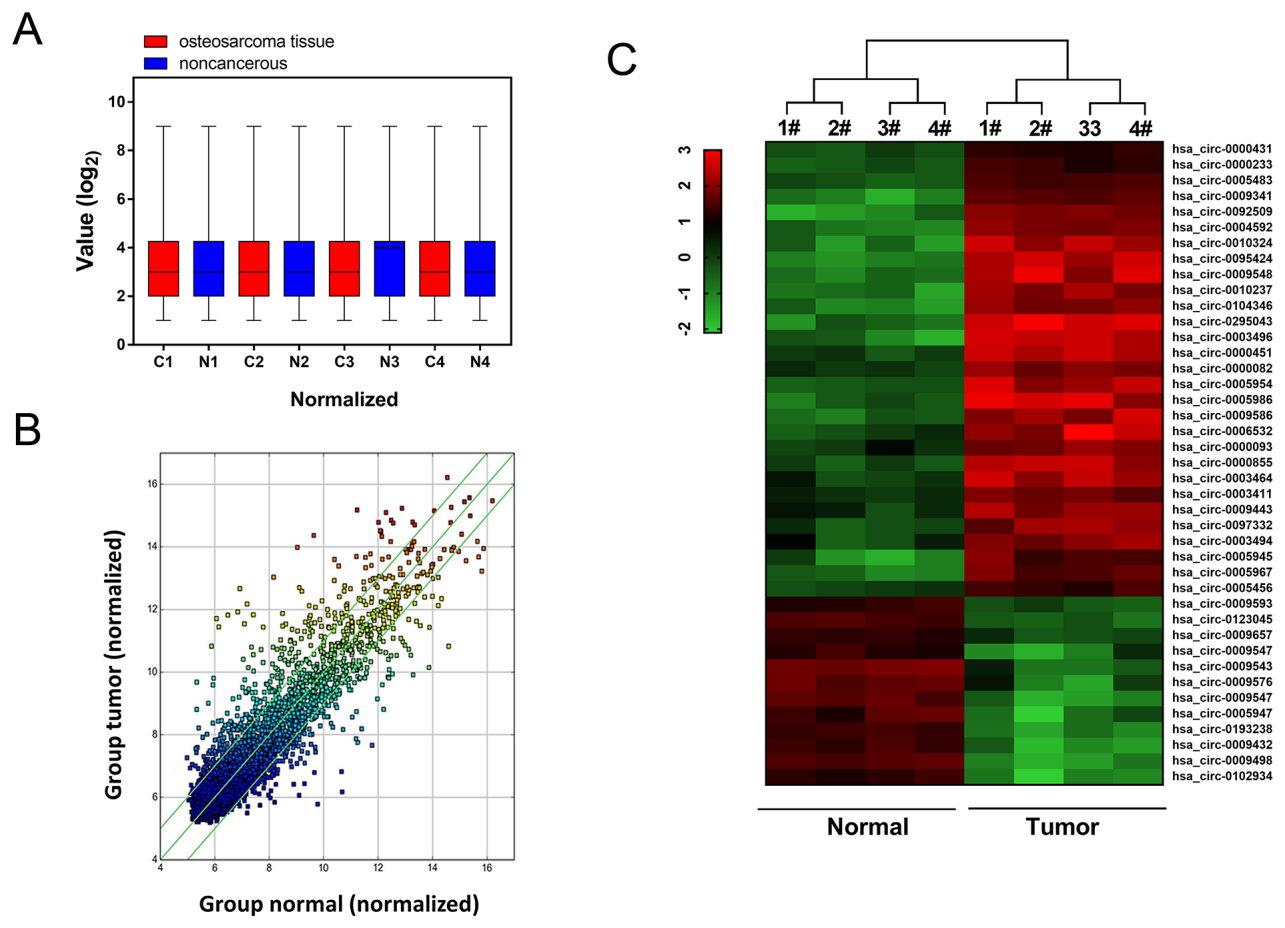
Circular RNA microarray expression profiles of osteosarcoma tissue and adjacent normal tissue **(A)** Box plot revealed the normalized intensities of 4 pairs of osteosarcoma tissue and adjacent normal tissue samples. **(B)** Scatter plot was performed to present the dysregulated circRNAs between osteosarcoma tissues and adjacent noncancerous tissues. **(C)** Heat map and hierarchical cluster analysis showed the circular RNAs expression in 4 pairs of osteosarcoma tissue and adjacent normal tissue.

In order to filtrate the differently expressed circRNAs in osteosarcoma tissue and adjacent normal tissue, high-throughput human circular RNA microarray was performed in 4 pairs of osteosarcoma tissue and adjacent normal tissue. With normalized intensities of osteosarcoma and normal tissue samples, results showed that 785 differently expressed circRNAs were distinguished with 2 fold change (Figure 1A). Scatter plot showed the aberrantly expressed circRNAs between osteosarcoma tissues and adjacent noncancerous tissues (Figure 1B). Hierarchical cluster analysis and heat map showed the differently expressed circRNAs, including up-regulated and down-regulated circRNAs (Figure 1C). Therefore, circular RNA microarray analysis discovered the distinguishable circRNAs expression profiles within osteosarcoma tissue and adjacent normal tissue.

### Circ-NT5C2 was up-regulated in osteosarcoma tissue

**Figure 2:**
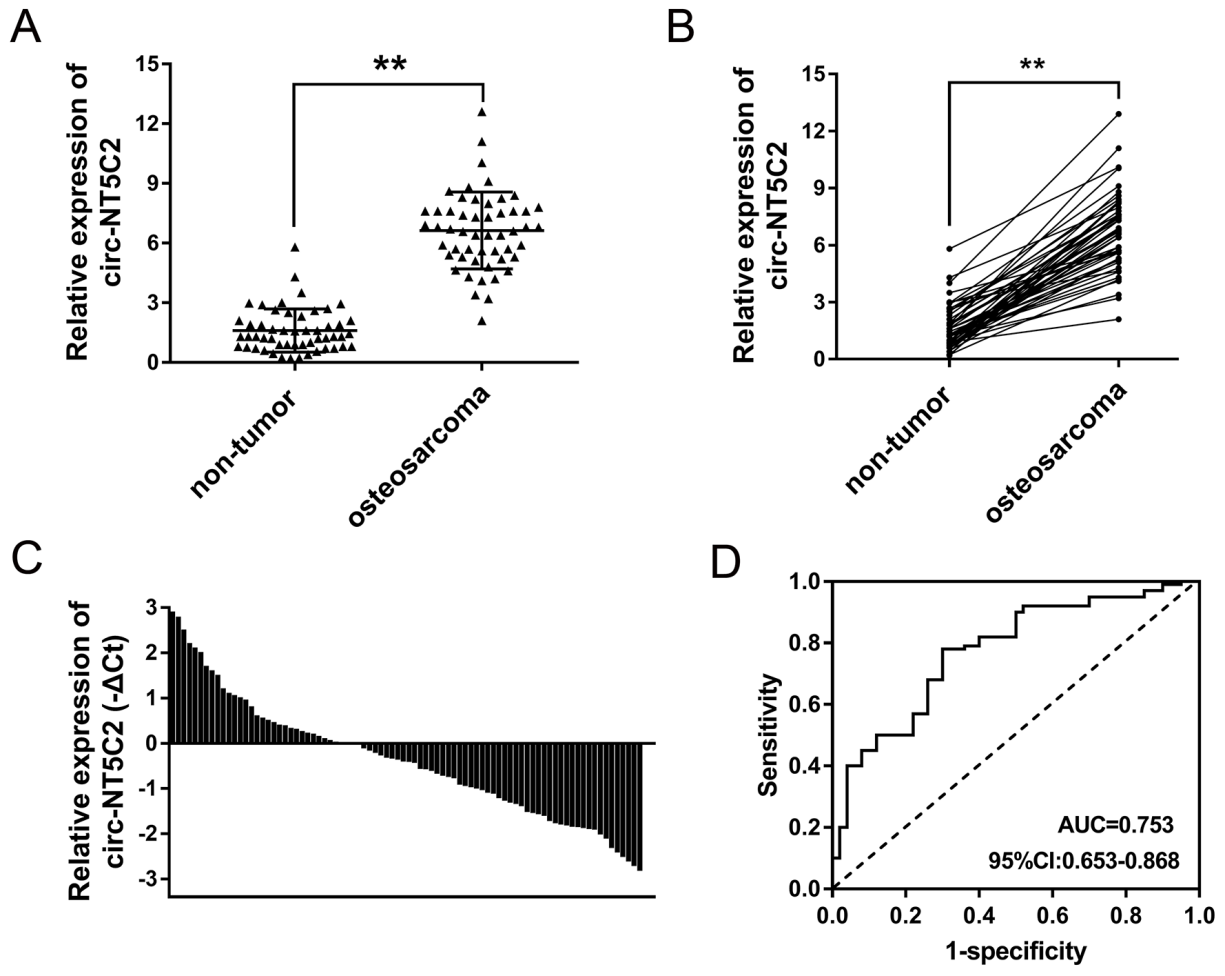
Circ-NT5C2 was up-regulated in osteosarcoma tissue **(A)** In 52 cases of osteosarcoma patients, circ-NT5C2 was significantly up-regulated in osteosarcoma tissue compared to adjacent non-tumor tissue. **(B)** Circ-NT5C2 was significantly up-regulated in osteosarcoma tissue. **(C)** Circ-NT5C2 levels were up-regulated in 36.5% (19/52) of osteosarcoma tissue samples. **(D)** Receiver operating characteristic (ROC) curve showed the diagnostic value of circ-NT5C2 in osteosarcoma tissue samples. The AUC (area under the curve) was 0.753. Data were expressed as mean ± SD. ^**^P<0.01 represents statistically difference.

By means of microarray analysis, we found that circ-NT5C2 (hsa_circ_0092509) was over-expressed in 4 pairs of osteosarcoma tissue. In further experiments, we validated the expression of circ-NT5C2 in 52 pairs of osteosarcoma tissue and adjacent normal tissue (Figure 2A, 2B). The basic clinical characteristics of these patients were documented in Table 1. Furtherly, circ-NT5C2 expression levels were up-regulated in 36.5% (19/52 cases of samples) of osteosarcoma tissue samples, and down-regulated in 63.5% (33/52) of that compared with adjacent non-tumor tissue (Figure 2C). Receiver operating characteristic (ROC) curve showed that, comparing with osteosarcoma patients and controls, circ-NT5C2 had an AUC (area under the curve) value of 0.753. Taken together, results revealed that circ-NT5C2 was up-regulated in osteosarcoma tissue compared with adjacent noncancerous tissue, acting as a valuable diagnostic marker for osteosarcoma detection.

**Table 1:** Relationship of circ-NT5C2 expression levels (ΔC_t_) in clinicopathological characteristics of osteosarcoma patients

characteristic		N (%)	Mean ± SD	P value
Gender	Male	31	14.634 ± 1.847	0.793
	Female	21	13.985 ± 2.042	
Age	<14	24	12.854 ± 1.985	0.627
	≥14	28	11.834 ± 1.784	
Chemotherapy	No	15	12.464 ± 2.063	0.028^*^
	Yes	37	14.311 ± 1.930	
Tumor size	<8cm	32	12.434 ± 2.049	0.132
	≥8cm	20	13.272 ± 2.303	
Tumor site	Femur/tibia	34	11.480 ± 1.984	0.689
	Elsewhere	18	12.094 ± 2.099	
Lung metastasis	No	40	13.954 ± 2.231	0.039^*^
	Yes	12	14.014 ± 2.093	
Enneking stage	I	16	12.857 ± 1.894	0.040^*^
	II	25	13.843 ± 2.355	
	III	11	14.935 ± 1.584	
Recurrence	No	29	12.583 ±1.695	0.078
	Yes	23	13.985±1.894	

^*^P<0.05 represents statistical differences.

### Circ-NT5C2 silencing suppressed osteosarcoma cells proliferation *in vitro*

**Figure 3:**
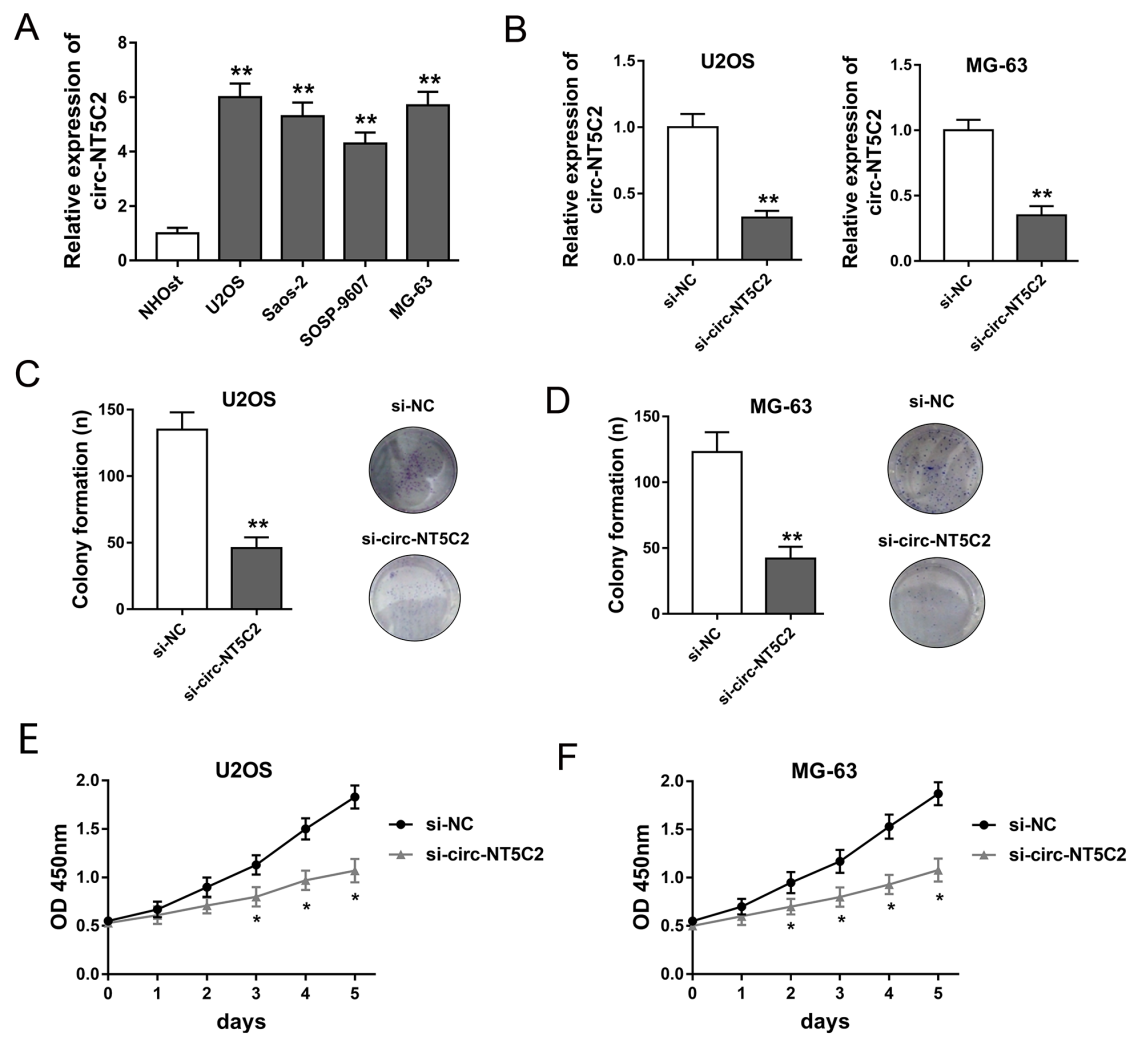
Circ-NT5C2 silencing suppressed osteosarcoma cells proliferation *in vitro* **(A)** Circ-NT5C2 expression detected by RT-PCR in osteosarcoma cell lines (MG-63, U2OS, SOSP-9607 and SAOS-2) and human osteoblastic cell line (hFOB). **(B)** Expression of circ-NT5C2 in U2OS and MG-63 cell lines transfected with siRNAs targeting circ-NT5C2. **(C, D)** Colony formation assay showed the clone number of U2OS and MG-63 cells. **(E, F)** CCK-8 assay showed the absorbancy of proliferation of U2OS and MG-63 cells. Data were expressed as mean ± SD. ^*^P<0.05, ^**^P<0.01 represents statistically difference.

Circ-NT5C2 had been tested to be increased in osteosarcoma tissue. *in vitro*, RT-PCR revealed that circ-NT5C2 expression levels were also significantly up-regulated in osteosarcoma cells compared with normal human osteoblastic cell line (Figure 3A). In U2OS and MG-63 cells, circ-NT5C2 expression is higher than other two cell lines. Therefore, the two cell lines are chosen as the target cell lines for loss-of-function experiments. Synthesized interfering oligonucleotides targeting circ-NT5C2 significantly decreased the expression of circ-NT5C2 in U2OS and MG-63 cell lines (Figure 3B). To assess the role of circ-NT5C2 on osteosarcoma cells proliferation, colony formation assay and CCK-8 assay were performed. Results showed that circ-NT5C2 silencing suppressed the osteosarcoma cells proliferation *in vitro*.

### Circ-NT5C2 silencing suppressed osteosarcoma cells progression *in vivo* and *in vitro*

**Figure 4:**
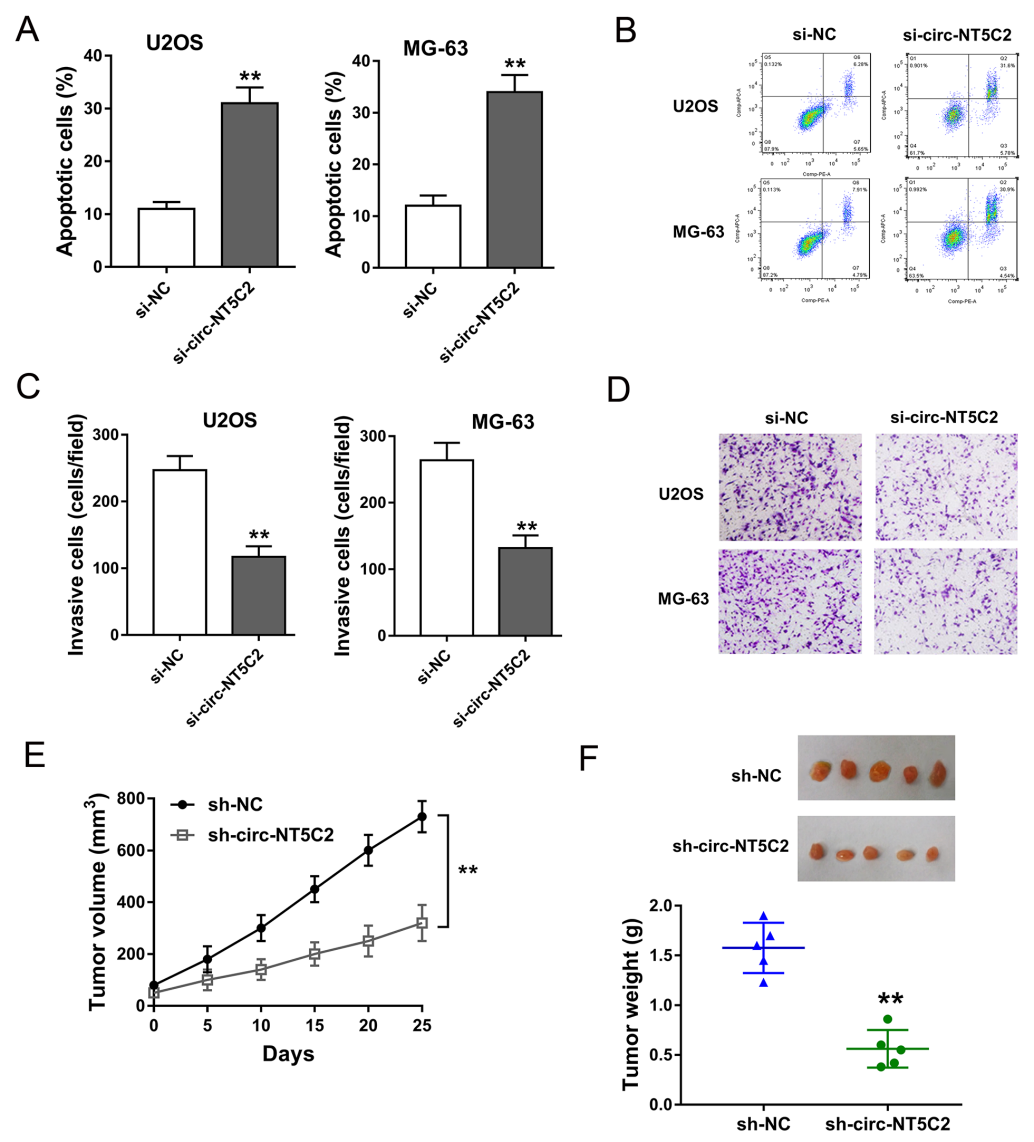
Circ-NT5C2 silencing suppressed osteosarcoma cells progression *in vivo* and *in vitro* **(A, B)** Apoptosis assay detected by flow cytometry showed the apoptotic cell rate in in U2OS and MG-63 cells transfected with si-circ-NT5C2 and negative controls. **(C, D)** Transwell invasive assay showed the invaded cells. **(E, F)** Xenograft mice assay *in vivo* showed the tumor volume and weight in nude mice injected U2OS cells transfected with sh-circ-NT5C2 or sh-NC. Data were expressed as mean ± SD. ^**^P<0.01 represents statistically difference.

To further investigate the function of circ-NT5C2 on osteosarcoma cells progression, more functional experiments were performed. Apoptosis assay detected by flow cytometry showed that, in U2OS and MG-63 cells, circ-NT5C2 silencing increased the apoptotic cell rate compared to negative control group (Figure 4A, 4B). Transwell invasive assay showed that circ-NT5C2 silencing suppressed the invaded cells compared to negative control group (Figure 4C, 4D). Xenograft mice assay in*vivo* showed that circ-NT5C2 knockdown not only suppressed the tumor volume, but also inhibited the tumor weight, suggesting the suppressor role of circ-NT5C2 knockdown on osteosarcoma tumor growth (Figure 4E, 4F). Overall, results indicated that circ-NT5C2 silencing could suppress the osteosarcoma cells progression *in vivo* and *in vitro*.

### Bioinformatics revealed that circ-NT5C2 sponged target miR-448

**Figure 5:**
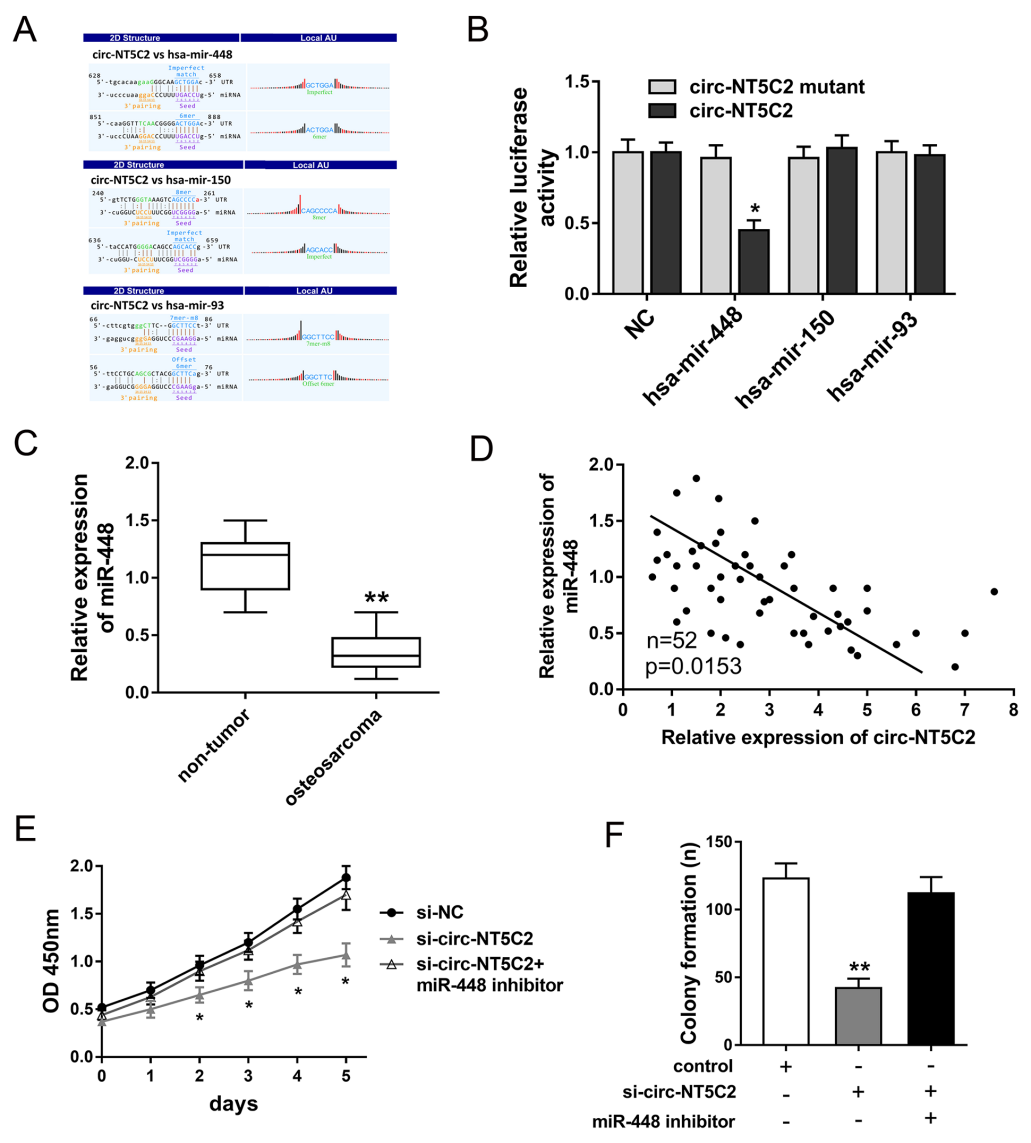
Bioinformatics analysis and experiments results manifested that circ-NT5C2 sponged miR-448 **(A)** The interaction analysis of circRNA/miRNA was predicted using Arraystar’s homemade software, basing on miRanda and TargetScan. **(B)** Luciferase reporter assay revealed the luciferase vitality in HEK293T cells when co-transfected with miRNAs mimics and circ-NT5C2 (wild type or mutant). **(C)** RT-PCR results revealed the miR-448 expression level in osteosarcoma tissue compared to adjacent non-tumor tissue. **(D)** Pearson’s correlation analysis showed the correlations between circ-NT5C2 and miR-448 expression in 52 cases of osteosarcoma tissue (P=0.0153). **(E)** CCK-8 assay showed the proliferation vitality of osteosarcoma cells (U2OS). **(F)** Colony formation assay showed the clone number of osteosarcoma cells. Data were expressed as mean ± SD. ^*^P<0.05, ^**^P<0.01 represents statistically difference.

Functional experiments had revealed the oncogenic role of circ-NT5C2 on osteosarcoma cells progression. Bioinformatics prediction online programs (starBase, miRBase, TargetScan) indicated that three miRNAs potentially targeted circ-NT5C2 UTRs, including hsa-mir-448, hsa-mir-150 and hsa-mir-93 (Figure 5A). Luciferase reporter assay indicated that luciferase vitality was decreased when co-transfected with miR-448 mimics and circ-NT5C2 wild type, while miR-150 and miR-93 didn't have the difference (Figure 5B). In osteosarcoma tissue, RT-PCR results revealed that miR-448 expression levels were significantly down-regulated compared to adjacent non-tumor tissue (Figure 5C). Pearson’s correlation analysis and long rank analysis showed the negatively association within the expression of circ-NT5C2 and miR-448 (Figure 5D). Moreover, CCK-8 assay and colony formation assay showed that miR-448 inhibitor could reverse the role of si-circ-NT5C2 on osteosarcoma cells (U2OS) proliferation (Figure 5E, 5F). Taken together, bioinformatics analysis and experiments results manifested that circ-NT5C2 sponged miR-448 and presented negatively association within circ-NT5C2 and miR-448.

## DISCUSSION

The huge amount of circular RNAs (circRNAs) have been discovered by next-generation sequencing methods or gene chip with the rapid development of high-throughput DNA technology in recent years [13, 14]. Following with long noncoding RNAs and miRNAs, literature about circRNAs have been rising with a large increase [15, 16]. Acting as a vital member of noncoding RNA, numerous circRNAs have been reported to participate in series of pathological process involved in osteosarcoma carcinogenesis [17].

In present study, our team performed human circRNAs microarray analysis in osteosarcoma tissue and adjacent noncancerous and found total 785 differently expressed circRNAs with 2 fold change. Finally, we identified circ-NT5C2 (hsa_circ_0092509, gene symbol NT5C2, www.circbase.org/). Then, circ-NT5C2 expression was verified to be up-regulated in 52 cases of osteosarcoma patient samples. Receiver operating characteristic (ROC) curve illustrates the diagnostic value of circ-NT5C2 for osteosarcoma patients detection (AUC=0.753). So much for that, microarray screening and clinical specimens analysis reveal the potential differently expressed circRNAs in osteosarcoma samples, and identify the enhanced expression of circ-NT5C2. By means of microarray or high-throughput sequencing, the most common methods for discovering the expression profiles of RNA, hundreds or thousands of circRNAs or lncRNAs are distinguished [18]. These RNA library from next-generation sequencing provide plentiful candidates for molecular mechanism researches [19]. For circular RNA PCR, our team designed the outward facing primers for circ-NT5C2 and synthesized it by Sangon Biotech (Shanghai, China). Because circular RNA is characterized by special covalently closed loop configuration, the primers for PCR are outward facing primers or divergent primers, instead of common line RNA.

Now that the differently circRNAs expression profiles elected the significantly expressed circ-NT5C2, the further functional validation assay were necessary to verify the potential physiological functions. *In vitro* assay, including CCK-8, colony formation assay, and invasion assay, showed the suppression of circ-NT5C2 knockdown on osteosarcoma cells proliferation and invasion. *In vivo*, circ-NT5C2 knockdown inhibited the tumor growth. Thus, not limited in the expression profiles screening, we also identified the biological function of one typical representative, suggesting the oncogenic role of circ-NT5C2 in osteosarcoma tumorigenesis.

Similarly, being similar to lncRNAs, circRNAs also have certain characteristics on the epigenetic regulation, involved in transcriptional regulation and post-transcriptional regulation [20]. The most famous circRNA is ciRS-7, which harbor about 70 miR-7 binding sites and regulate multiple cancers, including gastric cancer and hepatocellular carcinoma [21, 22]. In bladder carcinoma, circRNA-MYLK accelerated cell proliferation, migration, tube formation of HUVEC, and circRNA-MYLK knockdown decreased cell proliferation, motility, and induced apoptosis [23]. In present study, we found that circ-NT5C2 was up-regulated in osteosarcoma tissue and cells, and function as oncogenic molecular in the tumorigenesis, providing a valuable diagnostic marker and therapeutic target for osteosarcoma detection.

Emerging articles have been published to report the circular RNA expression and biological roles in osteosarcoma. For example, Li JF et al reported that cir-GLI2 was significantly upregulated in osteosarcoma tissues and cells, and exerted the tumor-promoting effects on osteosarcoma cells via negatively targeting miR-125b-5p [24]. Circular RNA hsa-circ-0016347 promotes proliferation, invasion and metastasis of osteosarcoma cells through acting as a positive regulator in osteosarcoma cells proliferation and invasion, and miR-214 regulates the expression of caspase-1 [25].

Although circular RNAs don't have the protein coding capacity, circRNAs exert their vital regulatory role via targeting miRNAs and functional gene mRNA. Up to now, one of the important regulatory mechanism is miRNA ‘molecular sponge’[26]. Our prior experiments and data reveal that circ-NT5C2 regulate the OS cells progression. Besides, as well-known, circular RNA might function as the miRNA ‘sponge’. Thus, bioinformatics tools illustrate that there are 3 candidate miRNAs, and ultimately, miR-448 has been verified as the functional miRNA that could reverse the role of circ-NT5C2 on OS tumorigenesis, suggesting the ‘sponge’ mechanism within circ-NT5C2 and miR-448. The pathway of circRNA-miRNAs-mRNA axis in human diseases has been wildly illustrated [27]. For example, in Hirschsprung’s disease, circRNA ZNF609 functions as a competitive endogenous RNA (ceRNA) to regulate AKT3 expression by sponging miR-150-5p [28]. In vascular endothelial cell, hsa_circ_0010729 co-express with HIF-1α and negatively correlated with miR-186 in hypoxia induced HUVECs progression [28]. In human keratinocytes, circ100284 is involved in the arsenite-accelerated cell cycle via miR-217 EZH2 regulation [29]. Thus, increasing evidence verify the significant value of circRNA-miRNAs-mRNA axis in pathogenesis.

In present study, our team investigates the circRNAs expression profiles in osteosarcoma tissue and determines the tumorous roles of circ-NT5C2 in osteosarcoma tumorigenesis. Circ-NT5C2 functions as an oncogenic molecular through targeting miR-448, acting as miRNA ‘sponge’, which provides novel insights for the osteosarcoma carcinogenesis.

## MATERIALS AND METHODS

### Human osteosarcoma clinical specimens

A total of 56 paired osteosarcoma and corresponding adjacent noncancerous tissues were collected at the Shenzhen People’s Hospital from May 2015 to Aug 2016. All tissues specimens were resected during surgery and directly snap frozen in liquid nitrogen. The study protocol was approved by the ethics committee of the Shenzhen People’s Hospital. All written informed consents had received from patient who underwent surgery resection.

### Microarray analysis

Total RNA was extracted from 4 pairs of osteosarcoma samples, and the enrichment of circRNA was increased after digesting with Rnase R (Epicentre, Inc.). Then, the RNA quantifying was performed using the NanoDrop ND-1000 (Thermo Scientific, Wilmington, DE, USA). The circular RNA microarray hybridization were performed according to Arraystar’s standard protocols. Briefly, total RNAs Then, the enriched circular RNAs were amplified and transcribed into fluorescent cRNA utilizing a random priming method (Arraystar Super RNA Labeling Kit; Arraystar). The labeled cRNAs were hybridized onto the Arraystar Human circRNA Array (8×15 K, Arraystar). After having washed the slides, the arrays were scanned by the Agilent Scanner G2505C

### Cell lines and cell culture and transfection

The human osteosarcoma cell lines (MG-63, U2OS, SOSP-9607 and SAOS-2) and human osteoblastic cell line (hFOB) were obtained from ATCC (Rockville, USA). All these cells were kept in Dulbecco’s modified Eagle’s medium (Gibco, Carlsbad, CA, USA) supplemented with 10 % fetal bovine serum (FBS, Gibco), streptomycin, and penicillin. circ-NT5C2 small-interfering RNA and miR-448 inhibitors were synthesized by RiboBio and transfected using Lipofectamine 2000 (Invitrogen, California, USA), according to the manufacturer’s instructions

### Quantitative RT-PCR

The extracted RNA from the cells was treated with Trizol reagent (Invitrogen, Carlsbad, Calif, USA). RT-qPCR analysis was performed with SYBR green in a 7500 Fast Real-Time PCR system (Applied Biosystems). For circRNAs, outward facing primers or divergent primers were designed and synthesized by Sangon Biotech (Shanghai, China). circ-NT5C2, 5′-AGTCCTAAGTTTTCCACTTCA-3′ (forward), 5′-AG GTGCCAGTAGCATTTTAGAC-3′ (reverse); miR-488, 5′-GCTGAGGGAGATATCGGCGCC-3′ (forward), 5′-GG AACACGCATGGCAGATCC-3′ (reverse); GAPDH, 5´-GCACCGTCAAGGCTGAGAAC-3´ (forward), 5´-TG GTGAAGACGCCAGTGGA-3′ (reverse). GAPDH was used as internal control for RNA expression. Samples were normalized to internal housekeeping genes followed by normalization of each sample to its control. The relative expression of candidate genes was calculated using the 2^−ΔΔCT^ equation. At least triple experiments were subjected to qRT-PCR verification

### Proliferation

CCK-8 assay and colony formation assay were performed to assess the proliferation of osteosarcoma cells. For CCK-8 assay, the mock and infected cells (1×10^5^ cells/well) were seeded into 96-well plates after 24h transfection and cultured for 7 days. The absorbance at 450 nm was measured using Cell Counting Kit-8 (Dojindo Laboratories, Japan) every day according to the manufacturer’s protocols. For colony formation assay, cells were placed in a fresh 6-well plate and maintained in 1640 medium containing 10% FBS. After 14 days, clones were fixed with 4% paraformaldehyde for 30 min and stained with 0.1% crystal violet. Visible colonies were manually counted

### Transwell invasion assay

For transwell invasion assay, osteosarcoma cells (5×10^4^) suspended in FBS free medium with 1 μg/ml Mitomycin C were plated in the upper well of a 24-well poly-carbonate transwell insert (Millipore, Bedford, MA, USA). The membrane in the upper chamber was coated with Matrigel (Beyotime Institute of Biotechnology, Haimen, China) 6 h prior to seeding. Cell invasion assays were performed using the Cell Invasion Assay Kit (ECM550, Millipore) following the manufacturer’s protocols. The lower chamber was filled with 600 μl medium containing 10% FBS. After 24h of incubating, the non-invaded cells were removed with a cotton swab. The outer membrane was fixed and stained. The invasion mages were gained under an inverted light microscope (Leica Microsystems, Wetzlar, Germany) at a magnification of x200

### Apoptosis assay

Flow cytometry was performed for the apoptosis analysis. osteosarcoma cells were digested with trypsin and washed with PBS. After blocking with 10% FBS, cells were stained with 5 μl FITC annexin-V and 1 μl propidiumiodide (Becton Dickinson, Heidelberg, Germany) and incubated at room temperature for 15 mins in the dark. Then, flow cytometric was performed (Life Technologies, Darmstadt, Germany). The cells were then analyzed with CellQuest software (BD Biosciences). The dead cells and the relative ratio of apoptotic cells was compared in each experiment

### Luciferase reporter assays

The sequences of circ-NT5C2 containing the predicted miR-448 binding sites were amplified by PCR, and cloned into pGL3 Vector (Promega, Madison, WI, USA), named as pGL3-circ-NT5C2-wild type and pGL3-circ-NT5C2-mutant. HEK 293T cells (5×10^3^ cells per well) were co-transfected with miR-448 mimics or blank controls, and pGL3-circ-NT5C2-wild type or pGL3-circ-NT5C2-mutant using Lipofectamine 2000 (Invitrogen, California, USA), according to the manufacturer’s instructions. HEK 293T cells were seeded into in 96-well plates for 24 hours culture. The luciferase activities were assessed using Dual-Luciferase Reporter Assay System (Promega) on the Promega GloMax 20/20 luminometer according to manufacturer’s protocol. The results were expressed relative to luciferase activity

### Xenograft model experiment

Ten male BALB/c nude mice (5-6 weeks) were purchased from Laboratory Animal Center of Shenzhen People’s Hospital and maintained under SPF conditions. The animal procedure was approved by the Committee on Animal Welfare of the Shenzhen People’s Hospital. Osteosarcoma cells (U2OS, 2×10^5^) transfected with sh-circ-NT5C2 or sh-NC subcutaneously injected into the flanks of mice. The tumors volume was measured every 5 days after the injection using calipers. Three weeks later, the mice were euthanized and tumor weight was tested

### Statistical analysis

All statistical analyses were performed using SPSS software (SPSS 16.0, Chicago, IL) and GraphPad Prism 6.0 software (GraphPad Software Inc., San Diego, CA, USA). Data were presented as mean ± standard deviation (SD). Statistical analysis was calculated using independent-samples t-test and one way ANOVA. P < 0.05 was considered as significant difference. Hierarchical cluster analysis was performed to group samples based on expression values. Receiver operating characteristic (ROC) analyses were performed using pROC package in R language. The area under the curve (AUC) under binomial exact confidence interval was calculated to generate the ROC curve

## Abbreviations

circRNAs: circular RNAs
ncRNAs: non-coding RNA
miRNA: micro RNA
ROC: receiver operating characteristic
AUC: area under the curve
ceRNA: competitive endogenous RNA
